# Dietary fiber during gestation improves lactational feed intake of sows by modulating gut microbiota

**DOI:** 10.1186/s40104-023-00870-z

**Published:** 2023-05-05

**Authors:** Shuang Li, Jie Zheng, Jiaqi He, Hao Liu, Yingyan Huang, Liansu Huang, Ke Wang, Xilun Zhao, Bin Feng, Lianqiang Che, Zhengfeng Fang, Jian Li, Shengyu Xu, Yan Lin, Xuemei Jiang, Lun Hua, Yong Zhuo, De Wu

**Affiliations:** Key Laboratory for Animal Disease-Resistance Nutrition of China Ministry of Education, Institute of Animal Nutrition, Sichuan Agricultural University, No. 211, Huimin Road, Wenjiang District, Chengdu, 611130 Sichuan People’s Republic of China

**Keywords:** Dietary fiber, *Escherichia-Shigella*, Feed intake, *Lactobacillus*, Sow

## Abstract

**Background:**

The feed intake of sows during lactation is often lower than their needs. High-fiber feed is usually used during gestation to increase the voluntary feed intake of sows during lactation. However, the mechanism underlying the effect of bulky diets on the appetites of sows during lactation have not been fully clarified. The current study was conducted to determine whether a high-fiber diet during gestation improves lactational feed intake (LFI) of sows by modulating gut microbiota.

**Methods:**

We selected an appropriate high-fiber diet during gestation and utilized the fecal microbial transplantation (FMT) method to conduct research on the role of the gut microbiota in feed intake regulation of sows during lactation, as follows: high-fiber (HF) diet during gestation (*n* = 23), low-fiber (LF) diet during gestation (*n* = 23), and low-fiber diet + HF-FMT (LFM) during gestation (*n* = 23).

**Results:**

Compared with the LF, sows in the HF and LFM groups had a higher LFI, while the sows also had higher peptide tyrosine tyrosine and glucagon-like peptide 1 on d 110 of gestation (G110 d). The litter weight gain of piglets during lactation and weaning weight of piglets from LFM group were higher than LF group. Sows given a HF diet had lower Proteobacteria*,* especially *Escherichia-Shigella,* on G110 d and higher *Lactobacillus,* especially *Lactobacillus_mucosae_LM1* and *Lactobacillus_amylovorus,* on d 7 of lactation (L7 d). The abundance of *Escherichia-Shigella* was reduced by HF-FMT in numerically compared with the LF. In addition, HF and HF-FMT both decreased the perinatal concentrations of proinflammatory factors, such as endotoxin (ET), lipocalin-2 (LCN-2), tumor necrosis factor-α (TNF-α), and interleukin-1β (IL-1β). The concentration of ET and LCN-2 and the abundance of Proteobacteria and *Escherichia-Shigella* were negatively correlated with the LFI of sows.

**Conclusion:**

The high abundance of Proteobacteria*,* especially *Escherichia-Shigella* of LF sows in late gestation, led to increased endotoxin levels, which result in inflammatory responses and adverse effects on the LFI of sows. Adding HF during gestation reverses this process by increasing the abundance of *Lactobacillus,* especially *Lactobacillus_mucosae_LM1* and *Lactobacillus_amylovorus*.

## Background

Lactation is a critical stage in the reproductive cycle of sows. During lactation, sows must not only maintain their nutritional needs, but also produce large amounts of milk to provide the essential nutrients for the growth and development of piglets. Some studies have indicated that sow feed intake during lactation is often lower than the sow needs [1]. Breed, diet, backfat thickness, and feeding management are the main factors that affect feed intake of sows during lactation.

In addition, the feed intake of sows during lactation is affected by the energy intake during gestation [2, 3]. Therefore, low energy in gestation and high energy in lactation feeding patterns are often used to improve lactation feed intake (LFI) of sows in modern pig production. The use of high dietary fiber (DF) not only achieves the purpose of low-energy intake during pregnancy, but also increases the satiety of sows during pregnancy, which is beneficial to animal welfare [4, 5]. Most studies have shown that a high fiber (HF) diet in gestation improves voluntary feed intake during lactation [2, 4, 6–8]. However, the mechanism underlying the effect of bulky diets on the appetites of sows during lactation have not been fully clarified. Recently, a growing body of research has shown that gut microbiota participates in the regulation of the host appetite. The gastrointestinal tract is teeming with numerous symbiotic microorganisms. The growth and proliferation of the gut microbiota depend on the feed intake of the host as a source of energy [9]. The complex relationship between the gut microbiota and metabolites produced by microbiota affects host energy metabolism [10]. Bacteria and the bioactive molecules after bacterial lysis, such as lipopolysaccharide (LPS) and some bioactive metabolites, activate enteroendocrine cells (EECs) directly or indirectly (via enterocytes) to release peptide tyrosine tyrosine (PYY) and glucagon-like peptide 1 (GLP-1), which results in satiety [11]. Moreover, it has been reported that *Escherichia-Shigella* secretes caseinolytic protease (ClpB), the homolog hormone of α-MSH [12] which can activate the anorexia pathway [13]. Previous studies have shown that the composition of gut microbiota is related to the metabolic phenotype of the host. Transplantation of obese microbiota can lead to obesity and hyperfeeding in the recipient, indicating that gut microbiota influence the feeding behavior of the host [14, 15]. Chagwedera et al. [16] reported that *Lactobacillus johnsonii* Q1-7 rescues body weight and food intake in Tsc1f/fCD11cCre mice. Additional studies have indirectly demonstrated the role of the gut microbiota in host eating behavior. Furthermore, sow enzymes cannot degrade DF, therefore sows need gut microbiota to utilize DF. DF, as the major energy source for gut microbiota, is thought to affect the composition and diversity of microbiota [17–19]. However, few studies have attempted to determine whether a HF diet during gestation improves the LFI of sows by modulating gut microbiota. Fecal microbial transplantation (FMT) is a direct means to study the effects of gut microbiota. The collective evidence has revealed the similarity between intestinal microbiota of recipients and donors, as well as the normalization of gut microbial composition and function in recipients after FMT therapy in humans [20, 21]. Recent studies have shown that the use of FMT improves diarrhea or growth performance of recipients in pigs [22, 23]. These studies indicated that FMT can be used to directionally restore the gut microbiota of sows and to focus research on the role of microbiota in regulating the LFI of sows.

HF feed is usually used in gestation to increase the voluntary feed intake of sows during lactation. Recent studies have shown that gut microbiota is involved in regulation of the host appetite. However, the role of gut microbiota in improving the LFI of sows fed a HF diet during gestation has not been established. Therefore, the present study selected an appropriate HF diet during gestation and utilized the FMT method to focus research on the role of gut microbiota in feed intake regulation of sows during lactation. Our results will provide some insights and ideas for DF supplementation of sows during gestation to improve feed intake of sows during lactation through microbial pathway.

## Materials and methods

The experiment followed the animal protection law (Ethic Approval Code: SCAUAC201308−2) and was performed in accordance with the Guide for the Animal Care and Use approved by Sichuan Agricultural University Institutional Animal Care and Use Committee.

### Animals, diets, and experimental design

A total of 69 Landrace × Yorkshire parity two sows with a similar body weight (BW, 199.53 ± 3.03 kg) and backfat (BF, 14.59 ± 0.55 mm) were used. Sows were inseminated with semen from the same Duroc boar. After insemination, sows were then allocated to one of three treatments according to their BW and BF. The three treatments were low-fiber (LF) diet, high-fiber (HF) diet, and LF diet with HF-FMT (LFM: Fecal microbiota was derived from sows on a HF diet) throughout gestation (Donor and recipient sows were maintained at the same gestational age). The LF and HF diets contained 12.08% or 34.38% dietary fiber, respectively. The compositions of these diets are shown in Table 1.

**Table 1:** The ingredient composition and nutrient levels of diets

Item	Gestation		Lactation
	LF	HF	
Ingredient, %			
Corn	79.59	48.41	62.89
Dehulled soybean meal	14.00	4.00	22.13
Wheat bran	-	13.50	6.00
Sugar beet pulp	-	18.50	
Soybean hulls	-	10.00	
Fish meal	1.50	1.50	2.60
Soybean oil	1.50	1.50	2.00
*L*-Lys HCl (98%)	0.08	0.17	0.27
*DL*-Met (99%)	-	0.02	0.13
*L*-Thr (98.5%)	0.08	0.14	-
*L*-Trp (98%)	-	0.02	-
Limestone	1.10	0.51	0.98
Dicalcium phosphate	1.20	0.84	1.50
Sodium chloride	0.40	0.34	0.40
Choline chloride(50%)	0.15	0.15	0.15
Vitamin and mineral premix	0.40^1^	0.40^1^	0.50^2^
Total	100.00	100.00	100.00
Calculated nutrient levels^3^			
DE, Mcal/kg	3.38	3.00	3.27
NE, Mcal/kg	2.52	2.12	-
Crude protein, %	14.05	11.82	17.50
Crude fat, %	4.59	4.19	
Soluble fiber, %	1.72	6.00	
Insoluble fiber, %	10.36	28.38	
Insoluble fiber/Soluble fiber	6.32	4.73	
Dietary fiber, %	12.08	34.38	
Calcium, %	0.88	0.74	0.90
Available phosphorus, %	0.38	0.32	0.90
SID-Lys, %	0.65	0.55	0.98
SID-Met, %	0.20	0.17	
SID-Thr, %	0.51	0.43	
SID-Trp, %	0.13	0.11	

*DE* Digestible Energy, *NE* Net energy, *HF* High fiber diet during gestation, *LF* Low fiber diet during gestation

^1^Mineral and vitamin premixes provided per kilogram of gestational diet: Fe, 120 mg; Cu, 20 mg; Mn, 60 mg; Zn, 120 mg; Se, 0.3 mg; I, 0.5 mg; vitamin A, 10,000 IU; vitamin D_3_, 2000 IU; vitamin E, 60 IU; vitamin K, 5.0 mg; vitamin B_1_, 5.0 mg; vitamin B_2_, 10.0 mg; vitamin B_6_, 6.0 mg; vitamin B_12_, 50 μg; Nicotinic acid, 40 mg; Pantothenic acid, 20 mg; Folic acid, 2.0 mg

^2^Mineral and vitamin premixes provided per kilogram of lactational diet: Fe, 120 mg; Cu, 20 mg; Mn, 30 mg; Zn, 120 mg; Se, 0.3 mg; I, 0.3 mg; vitamin A, 6000 IU; vitamin D_3_, 1200 IU; vitamin E, 50 IU; vitamin B_1_, 1.0 mg; vitamin B_2_, 3.6 mg; vitamin B_6_, 1.8 mg; vitamin B_12_, 12.5 μg; Nicotinic acid, 20 mg; Pantothenic acid, 12.5 mg; Folic acid, 2.0 mg

^3^Calculated according to Chinese Feed Database (2018) http://www.chinafeeddata.org.cn/slcfb-pdf/2018-01.pdf

Five sows from the HF group were selected and fresh feces was collected from these sows each week. These fresh fecal samples were used to prepare fecal suspensions in the FMT experiments. The fecal suspension was prepared using the protocol previously described [24]. Briefly, fecal samples were homogenized in sterile saline solution, then passed through 2.0-, 1.0-, 0.5- and 0.25-mm steel strainers (sterilized) in turn to remove the larger and small particles. Finally, the suspension sample was resuspended after centrifugation at 6,000 × *g* for 15 min at 4 °C and the fecal microbial suspension was obtained. We used nutrient broth plate medium to count the live microbes in the slurry. Subsequently, sterile glycerol was added to the slurry at a final concentration of 10%, and then the slurry was stored in liquid nitrogen.

**Fig. 1:**
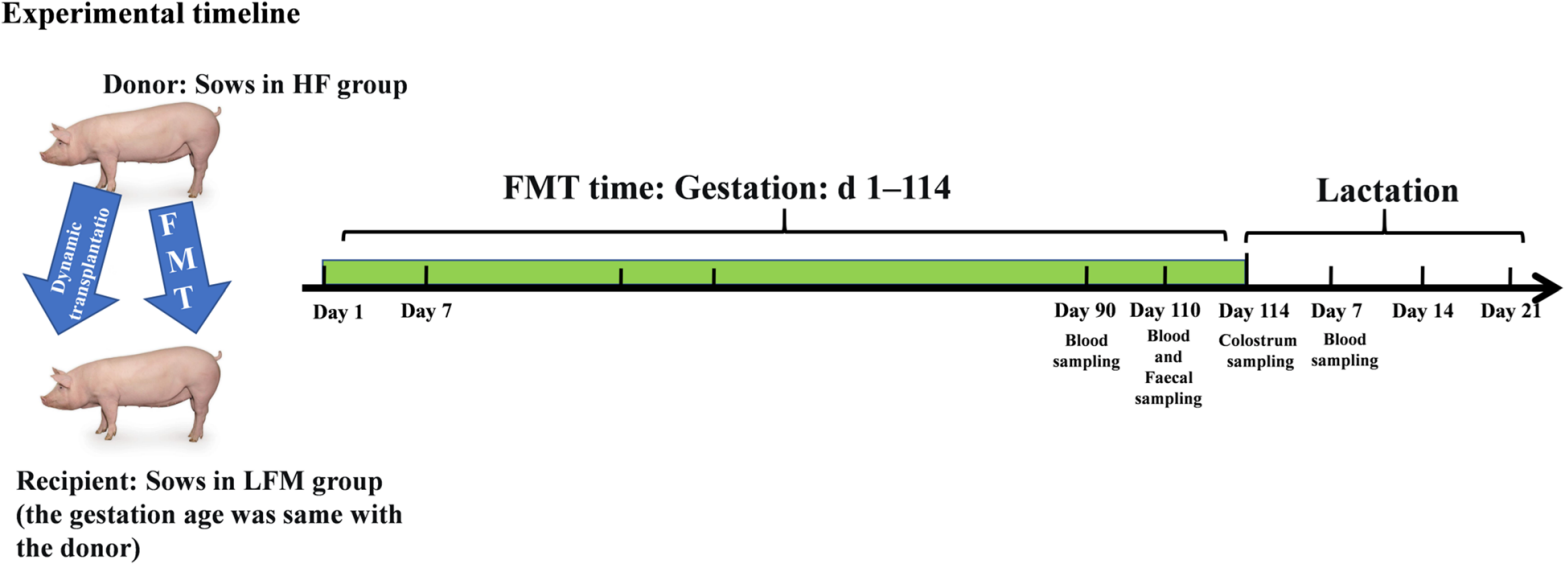
Schematic of experimental timeline. Five sows from the HF group were selected and fresh feces was collected from these sows each week. The sows in the LFM group with the same gestation age as the donor sows received a fecal suspension (10 mL [10^8^ CFU/mL]) containing fecal microbes by oral administration at 08:30 every day during gestation

All sows received two daily meals (at 08:00 and 14:00) during gestation. Sows were fed individually. The daily feed allowance (Table 2) was calculated to provide the same amount of net energy (NE) and crude protein (CP). The sows in the LF and HF diet groups received a vehicle (sterile saline) by oral gavage at 08:30 every day during gestation instead of the fecal suspension. The sows in the LFM group received a fecal suspension (10 mL [10^8^ CFU/mL]) containing fecal microbes by oral administration at 08:30 every day during gestation. A schematic showing the FMT inoculum preparation from the feces of HF diet sows and the frequency of transplantation are shown in Fig. 1. During lactation, the feeding amount was gradually increased from parturition to d 6 after parturition. Then the sows had free access to the diet afterwards. No creep feed was provided to the piglets during lactation. The dietary ingredients and composition of the lactation diet are shown in Table 1. The study began with 69 sows; 1 sow in the HF diet group and 2 sows in LF diet group were eliminated due to limb and hoof diseases after mating. The final number of pregnant sows used for the analysis was 66 and the final number of lactating sows used for analysis was 60 because 6 sows were eliminated due to disease or death.

**Table 2:** Daily feed allowances of pregnant sows

Item	Day of gestation, d		
	0–30	30–90	90–farrowing
LF, kg/d	2.50	2.30	2.70
LFM, kg/d	2.50	2.30	2.70
HF, kg/d	2.98	2.73	3.21

*HF* High fiber diet during gestation, *LF* Low fiber diet during gestation, *LFM* Low fiber diet + fecal microbiota transplantation from HF sow during gestation

### Measurement

The fasting BW and BF of sows were measured on d 0, 30, 60, 90, and 110 of gestation, farrowing day, and d 21 of lactation. BF was measured 65 mm to the left side of the dorsal mid-line at the level of the last rib (P2) using ultrasound (Renco Lean-Meatier; Renco Corporation, Minneapolis, MN, USA). After parturition, the total number of pigs born, living, mummified, stillborn, and low birth weight piglets (birth weight < 1,000 g) for each sow was recorded. The piglet birth interval and birth weight were recorded. In addition, the intra-litter coefficient of variation (CV) was calculated according to the piglet birth weight. Within 24 h after farrowing, litters were standardized to 12 piglets by cross-fostering within treatment groups. The daily feed intake of sows during lactation was recorded daily, and the individual weights of piglets and total number of piglets per litter were recorded at weaning. After weaning, estrus detection was performed once daily and the weaning-to-estrus interval (WEI) was recorded after estrus confirmation by standing heat in the presence of a boar.

### Sample collection

Blood samples (10 mL) were collected from the ear veins of sows on G110 d, L7 d, and L14 d after an overnight fasting period. Plasma samples were obtained by centrifuging blood samples at 3,000 × *g* for 15 min at 4 °C. The samples were immediately stored at −20 °C for further analysis.

Fresh feces were collected by massaging the rectum of sows on G110 d and L7 d. The fecal samples were then stored in liquid nitrogen.

### Metabolic biomarker analyses

The concentrations of endotoxin (ET) and lipocalin-2 (LCN-2) in plasma and feces were analyzed using the enzyme-linked immunosorbent assay kits (Jiangsu Meimian Industrial Co., Ltd., Jiangsu, China) according to the manufacturer’s instructions. The concentrations of PYY, glucagon-like peptide-1(GLP-1), secretory immunoglobulin A (sIgA), tumor necrosis factor-α (TNF-α), interleukin-1β (IL-1β), interleukin-10 (IL-10) and interleukin-6 (IL-6) in plasma were analyzed using the enzyme-linked immunosorbent assay kits (BIM Biosciences, Inc. San Francisco, USA) according to the manufacturer’s instructions.

### Short-chain fatty acid (SCFA) analysis

The concentrations of SCFAs, including acetic acid, propionic acid, and butyric acid in fecal samples, were determined by CP-3800 gas chromatography (Varian, Inc., Palo Alto, CA, USA) according to the improved method [25]. Approximately 0.7 g of fecal samples were thawed and diluted with 1.5 mL of ultrapure water, and 1.0 mL supernatant was obtained by centrifuging at 3,000 × *g* for 15 min. Then the supernatant was mixed with 0.2 mL of 25% metaphosphoric acid solution and 23.3 μL of 210 mmol/L crotonic acid and the mixed solution was placed at 4 °C for 30 min before centrifuging at 4,000 × *g* for 10 min, afterwards the 0.3 mL of supernatant was mixed with 0.9 mL of methanol, filtered by 0.22 μm filter (Millipore Co., Bedford, MA, USA) after centrifuging at 3,500 × *g* for 5 min.

### DNA extraction and PacBio sequencing of bacterial 16S rRNA gene from fecal microbiome species

Microbial community genomic DNA was extracted from fecal samples collected on G110 d (the sample size was 21, 23, 22 in LF, LFM and HF group respectively) and L7 d (the sample size was 15, 18, 19 in LF, LFM and HF group respectively, feces sample from eight sows were not collected) using the E.Z.N.A. soil DNA kit (Omega Bio-tek, Norcross, GA, USA) according to manufacturer’s instructions. DNA concentration and purity were determined with NanoDrop 2000 UV–vis spectrophotometer (Thermo Scientific, Wilmington, USA). The hypervariable region V3-V4 of the bacterial 16S rRNA gene were amplified with primer pairs 338F (5'-ACTCCTACGGGAGGCAGCAG-3') and 806R(5'-GGACTACHVGGGTWTCTAAT-3') by an ABI GeneAmp 9700 PCR thermocycler (ABI, CA, USA). Sequenced on an Illumina MiSeq PE300 platform (Illumina, San Diego, USA) according to the standard protocols by Majorbio Bio-Pharm Technology Co., Ltd. (Shanghai, China), which generated 300 bp single-end reads.

### Statistical analysis

#### Reproductive performance data, hormone, and SCFA data analysis

All calculations and statistical analyses were performed using SAS software (SAS 9.4; SAS Institute, Inc., Cary, NC, USA) with the individual sow as the experimental unit. Before parametric analysis, descriptive statistics were performed to check the normality and homogeneity of variance. The UNIVARIATE procedure was used to test residuals for outliers. Normality checks were carried out using PROC UNIVARIATE with NORMAL and PLOT options. The total number of piglets born, piglets born alive, piglets born alive weighing < 1,000 g, litter size after cross-fostering, litter size at weaning, and the WEI were analyzed using the GLIMMIX procedure with the Poisson distribution. The BW and BF loss of sows during lactation, fecal and plasma hormone concentration data, and fecal SCFA concentration data were analyzed using the MIXED procedure fitted assuming a normal distribution with DDFM = KR options included in the following model: *Y*_*i*_ = *μ* + *α*_*i*_ + *ε*_*i*_, in which *Y* is the analyzed variable; *μ* is the mean; *α*_*i*_ is the effect of diets (*i* = 1, 2, or 3); and *ε*_*i*_ represents the residual error. The individual piglet weight, litter weight, individual piglet weight gain and litter weight gain data during lactation were analyzed using the MIXED model analysis of covariance, litter size at the same time point was used as covariate in the model. The model was: *Y*_*i*_ = *Trt*_*i*_ + *slopeiX*_*i*_ + *error*_*i*_, *i* = 3 treatments and *X* = litter size. The litter size and average daily feed intake (ADFI) of sows every week during lactation using the SAS MIXED procedure for repeated measurements. Before analysis, the best covariance assumption structures model (SIM, CS, AR [1], ANTE [1], UN, and CSH) was selected based on the Akaike and Bayesian information criteria values. Multi-comparison was conducted by the Tukey test. The correlations of plasma hormone and bacterial abundance with lactational feed intake of sows were analyzed using the MIXED procedure for correlation analysis. Data are displayed as least squares means and a pooled SEM of each treatment, unless otherwise stated. *P* < 0.05 indicated a significant difference, while 0.05 ≤ *P* < 0.1 indicated a trend.

#### Microbiota data analysis

The unweighted UniFrac method was used to draw the principal coordinate analysis (PCoA) plots to visualize the differences in bacterial community composition among samples. Non-parametric analyses (analysis of similarity [ANOSIM]) for multivariable data were performed using the “WGCNA,” “stats,” and “ggplot2” package in R (version 2.15.3) for bacterial community structure comparison. The differences in relative abundance of bacteria between groups were analyzed by a Kruskal–Wallis H test bar plot analysis and Wilcoxon rank-sum test. Fixed effects in the model were dietary treatment and different stage of sows.

## Results

### Performance of sows

**Fig. 2:**
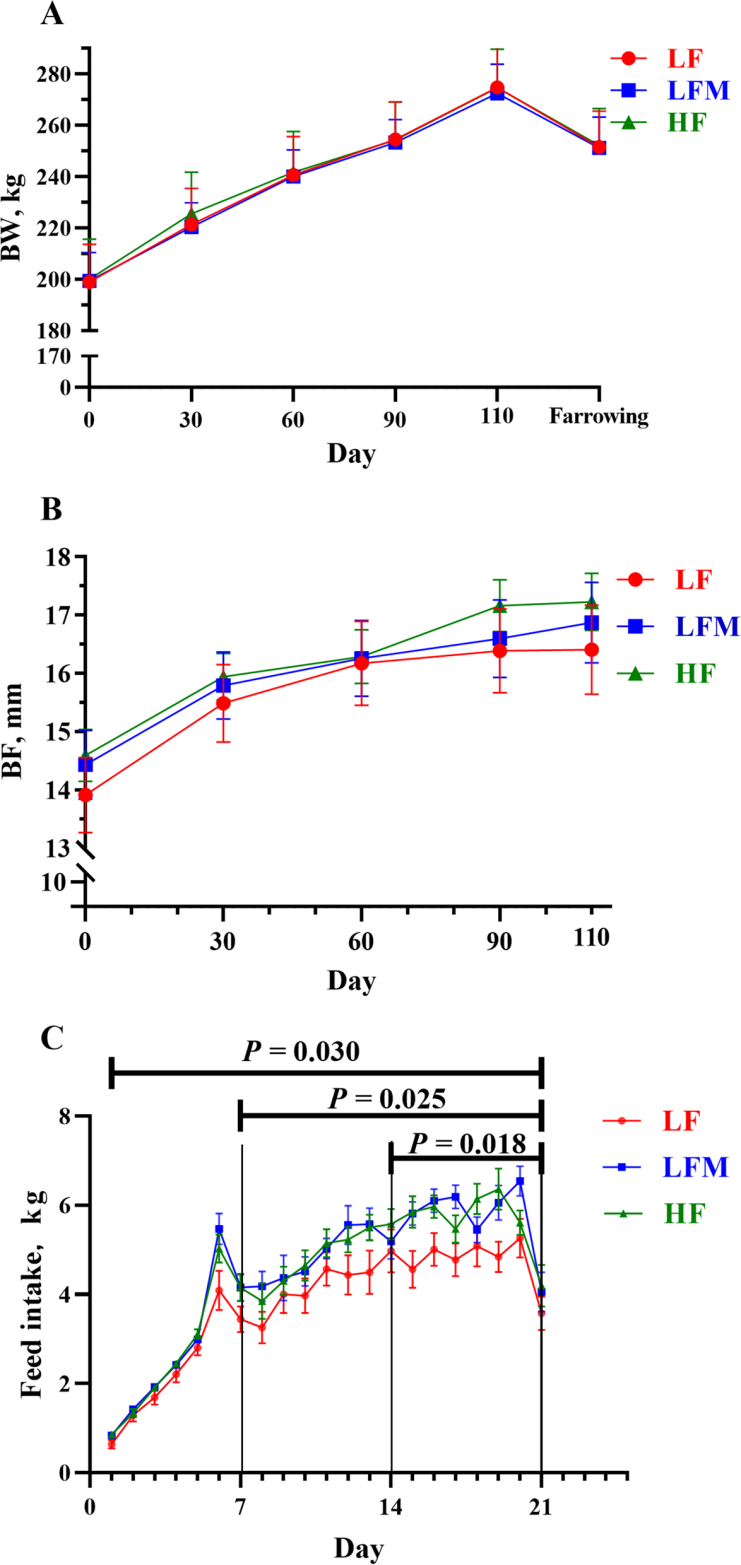
Effects of high fiber diet and HF-FMT during gestation on (**A**) body weight (BW) and (**B**) backfat (BF) changes during gestation and (**C**) daily voluntary feed intake of lactating sows. LF, low fiber diet during gestation; LFM, low fiber diet + fecal microbiota transplantation from HF sow during gestation; HF, high fiber diet during gestation; Data were expressed as least squares mean ± SEM. Sows were regarded as the experimental units, (**A** and **B**): LF, *n* = 21; LFM, *n* = 23; HF, *n* = 22. (**C**): *n* = 20 for each treatment

During the entire gestation, sows from all groups consumed their daily feed completely and no feed residue was recorded. The changes in sow BW and BF during gestation are shown in Fig. 2A and B. From mating to parturition, BW and BF thickness did not differ (*P* > 0.05) among treatments at any time point. During lactation, sows from the three groups lost the same amount of BW and BF (Table 3); however, the daily feed intake of sows varied during lactation (*P* < 0.05; Fig. 2C). Specifically, sows in the HF and LFM diet groups had higher feed intake than sows in the LF diet group (Fig. 2C). The difference in feed intake was significant during d 8–21 and d 15–21 of lactation and whole lactation (*P* = 0.025, *P* = 0.018 and *P* = 0.030, respectively; Fig. 2C). The WEI was not significantly different among the three groups (Table 3).

**Table 3:** Effects of high fiber diet and HF-FMT during gestation on performance of lactation sows

Item	Treatment			SEM	*P*-value
	LF	LFM	HF		
Number of sows, *n*	20	20	20	-	-
Body weight (BW), kg					
D 1 of lactation	249.20	248.18	252.46	3.269	0.640
D 21 of lactation	230.17	225.92	230.68	3.875	0.633
BW loss during lactation, kg	20.63	23.89	24.45	2.391	0.378
Backfat thickness (BF), mm					
D 1 of lactation	16.54	17.20	17.24	0.652	0.718
D 21 of lactation	13.79	13.82	14.55	0.725	0.617
BF loss during lactation, mm	2.69	3.30	2.53	0.316	0.232
WEI, d	6.25	5.33	5.08	0.272	0.337

*HF* High fiber diet during gestation, *n* = 20, *LF* Low fiber diet during gestation, *n* = 20, *LFM* Low fiber diet + fecal microbiota transplantation from HF sow during gestation, *n* = 20, *SEM* Pooled standard error of means, *BW* Body weight, *BF* Backfat, *WEI* Weaning-to-estrus interval

Data were shown as least squares mean with their SEM. Values within a row with different superscripts differ (*P* < 0.05)

### Reproductive performance of sows and performance of piglets

As shown in Table 4, no effect of DF and FMT during gestation was found with respect to the total number of piglets, piglets born alive, stillborn piglets, mummified fetuses, and individual BW for piglets born alive (*P* > 0.05). A HF diet during gestation tended to decrease the number of low birth weight piglets (*P* = 0.072), within-litter birth weight CV of piglets born alive (*P* = 0.096) and duration of parturition (*P* = 0.078). In addition, from standardization of litter size (24 h postpartum) to weaning, piglets born from LFM diet sows had heavier BW at weaning, litter weight at d 7 of lactation and litter weight gain at 1^st^ week of lactation than piglets from LF diet sows (*P* = 0.048, *P* = 0.027 and *P* = 0.024, respectively; Table 5). And FMT during gestation tended to increase the litter weight on d 14 and 21 of lactation (*P* = 0.083 and *P* = 0.089, respectively). The litter weight gain and the piglet mean average daily gain (ADG) from d 1 to 21 of lactation both were increased in LFM group compared with LF group (*P* = 0.016 and* P* = 0.031, respectively; Table 5). The litter size was not significantly different among the three groups (Table 5).

**Table 4:** Effects of high fiber diet and HF-FMT during gestation on reproductive performance of sows

Items	Treatment			SEM	*P*-value
	LF	LFM	HF		
Litters, *n*	21	23	22		
Total born, *n*	15.05	15.14	15.10	0.304	0.993
Born alive, *n*	13.80	14.14	14.40	0.273	0.722
Stillborn, *n*	1.33	1.00	1.00	0.039	0.437
Mummified fetuses, *n*	1.43	1.33	2.00	0.082	0.293
Low birth weight piglet^1^, *n*	2.17	1.50	1.36	0.061	0.072
Piglet weight at birth, kg	1.49	1.52	1.52	0.046	0.866
Litter weight at birth, kg	21.40	22.15	22.19	0.510	0.741
CV_bw_^2^, %	21.27	17.10	16.84	1.709	0.096
Duration of farrowing^3^, min	230.00	172.57	167.44	18.299	0.078

Data were shown as least squares mean with their SEM. Values within a row with different superscripts differ (*P* < 0.05)

*LF* Low fiber diet during gestation, *n* = 21, *LFM* Low fiber diet + fecal microbiota transplantation from HF sow during gestation, *n* = 23, *HF* High fiber diet during gestation, *n* = 22, *SEM* Pooled standard error of means

^1^Low birth weight piglet: Piglets with low birth weight (< 1,000 g)

^2^CV_bw_%: the intra litter coefficient of variation

^3^Duration of farrowing: defined as the time interval between birth of first and last piglet

**Table 5:** Effects of high fiber diet and HF-FMT during gestation on growth performance of lactation piglets

Items	Treatment			SEM	*P*-value		
	LF	LFM	HF		Trt	Time	Trt × time
No. of observations	20	20	20	-	-		
Litter size, *n*							
No. of piglets after cross-foster, *n*/litter	12.89	12.61	12.68	0.13	0.830		
D 7 of lactation	12.42	12.69	12.59	0.25	0.533	< 0.001	0.444
D 14 of lactation	11.89	12.14	12.11				
D 21 of lactation	11.52	12.09	11.88				
BW of piglets, kg							
BW of piglets after cross-foster, kg/head	1.50	1.52	1.55	0.04	0.762		
D 7 of lactation	2.52	2.70	2.61	0.08	0.216		
D 14 of lactation	3.86	4.28	4.08	0.13	0.074		
D 21 of lactation	5.40^b^	5.96^a^	5.60^ab^	0.16	0.048		
Litter weight, kg							
Litter weight after cross-fos ter, kg	19.02	19.52	19.88	0.54	0.528		
D 7 of lactation	30.63^b^	33.97^a^	33.82^ab^	0.97	0.027		
D 14 of lactation	46.41	51.58	49.09	1.61	0.083		
D 21 of lactation	64.14	70.31	67.08	1.93	0.089		
Litter weight gain, kg							
1^st^ week of lactation	11.62^b^	14.46^a^	13.95^ab^	0.76	0.024		
2^nd^ week of lactation	14.18	17.83	16.72	1.45	0.194		
3^rd^ week of lactation	17.79	19.81	19.04	0.80	0.200		
D 1 to 21 of lactation	42.47^b^	52.09^a^	49.64^ab^	2.45	0.016		
Piglet mean ADG (g/d)							
1^st^ week of lactation	147.83	162.63	156.21	6.05	0.226		
2^nd^ week of lactation	191.87	224.42	209.37	12.19	0.172		
3^rd^ week of lactation	220.18	240.34	217.33	9.22	0.166		
D 1 to 21 of lactation	185.70^b^	209.46^a^	193.34^ab^	6.33	0.031		

Data were shown as least squares mean with their SEM. ^a,b^Values within a row with different superscripts differ (*P* < 0.05)

*LF* Low fiber diet during gestation, *n* = 20, *LFM* Low fiber diet + fecal microbiota transplantation from HF sow during gestation, *n* = 20, *HF* High fiber diet during gestation, *n* = 20, *SEM* Pooled standard error of means

### Sow endocrine status

The results presented in Table 6, compared with LF group, the plasma concentrations of PYY and GLP-1 were significantly increased in HF diet sows on G110 d (*P* < 0.001 and *P* < 0.001, respectively) and L7 d (*P* = 0.002 and *P* < 0.001, respectively). A HF diet in gestation had no significant influence on the plasma PYY concentrations of sows on L14 d, but had a tendency to decrease the GLP-1 concentration (*P* = 0.050). Moreover, the plasma concentration of GLP-1 was higher in LFM group sows than LF group sows on G110 d (*P* < 0.001). However, there was no difference in PYY between LFM and LF group.

**Table 6:** Effects of high fiber diet and HF-FMT during gestation on plasma PYY and GLP-1 concentrations of sows

Items	Treatment			SEM	*P*-value
	LF	LFM	HF		
Plasma PYY, pmol/L					
110 d of gestation	3.79^c^	4.32^b^	5.02^a^	0.076	< 0.001
7 d of lactation	3.71^b^	3.95^b^	4.59^a^	0.087	0.002
14 d of lactation	4.19	3.93	3.93	0.108	0.204
Plasma GLP-1, pmol/L					
110 d of gestation	2.89^b^	3.71^a^	4.11^a^	0.066	< 0.001
7 d of lactation	2.86^b^	3.07^b^	3.66^a^	0.071	0.001
14 d of lactation	4.72	4.72	4.30	0.120	0.050

Data were shown as least squares mean with their SEM. Values within a row with different superscripts differ (*P* < 0.05)

*LF* Low fiber diet during gestation, *n* = 12, *LFM* Low fiber diet + fecal microbiota transplantation from HF sow during gestation, *n* = 12, *HF* High fiber diet during gestation, *n* = 12, *SEM* Pooled standard error of means, *PYY* Peptide YY, *GLP-1* Glucagon-like-peptide-1

**Fig. 3:**
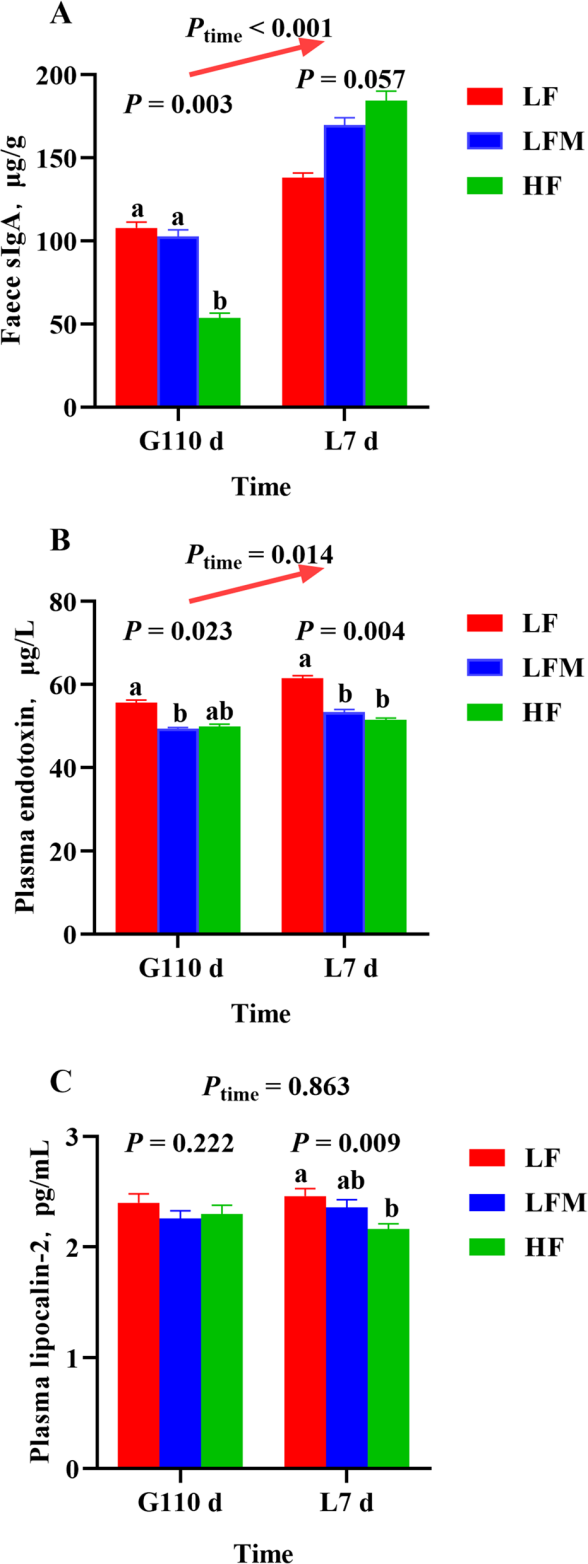
Effects of high fiber diet and HF-FMT during gestation on (**A**) fecal secretory immunoglobulin A (sIgA), (**B**) plasma endotoxin (ET) and (**C**) plasma lipocalin-2 (LCN-2) concentrations of sows. LF, low fiber diet during gestation; LFM, low fiber diet + fecal microbiota transplantation from HF sow during gestation; HF, high fiber diet during gestation; G110 d, d 110 of gestation; L7 d, d 7 of lactation; Data were expressed as least squares mean ± SEM (*n* = 10). Different letters denote significant differences (*P* < 0.05)

**Fig. 4:**
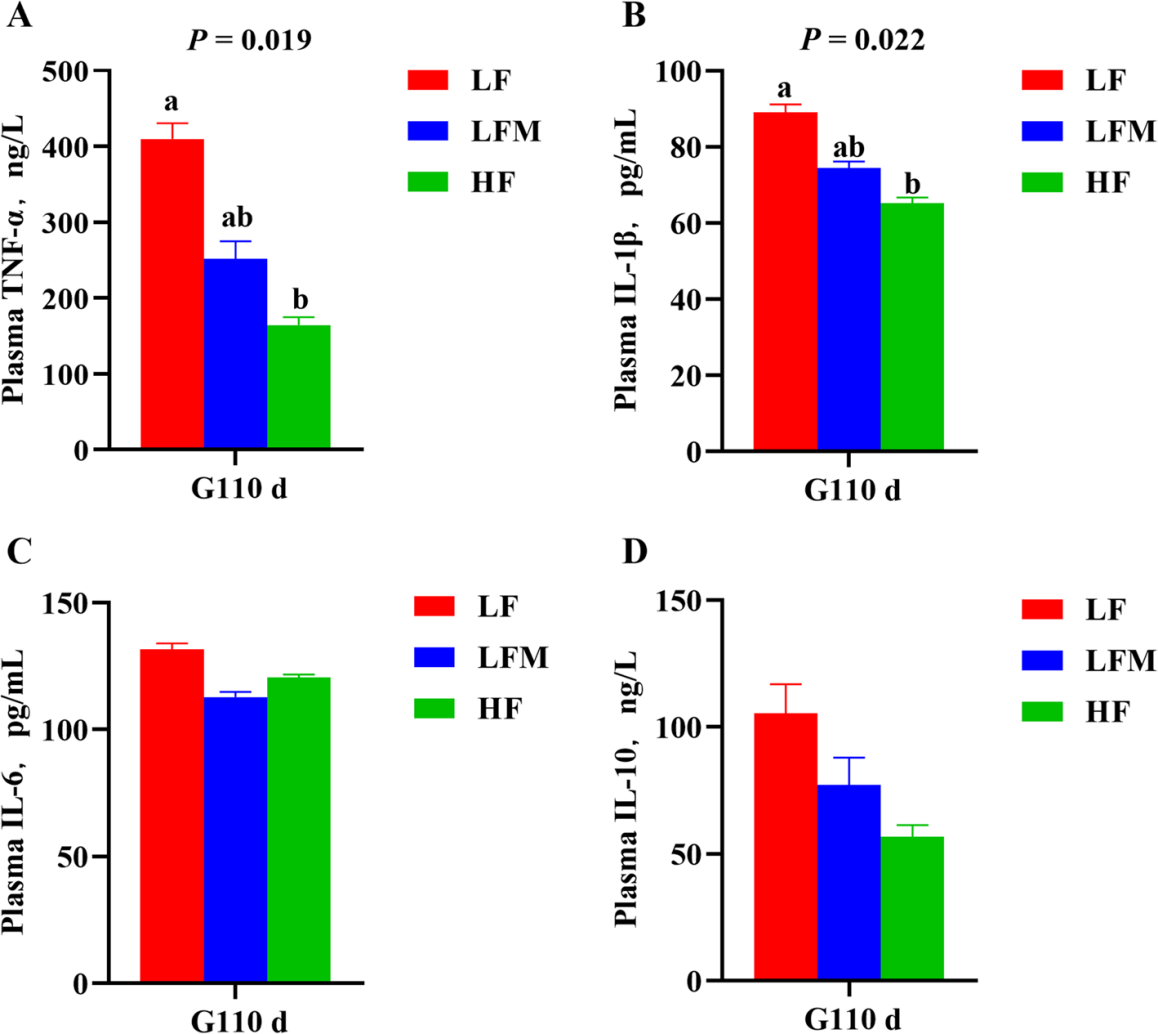
Effects of high fiber diet and HF-FMT during gestation on (**A**) tumor necrosis factor-α (TNF-α), (**B**) interleukin-1β (IL-1β), (**C**) interleukin-6 (IL-6) and (**D**) interleukin-10 (IL-10) in plasma of sows on G110 d. LF, low fiber diet during gestation; LFM, low fiber diet + fecal microbiota transplantation from HF sow during gestation; HF, high fiber diet during gestation; G110 d, d 110 of gestation; Data were expressed as least squares mean ± SEM (*n* = 10). Different letters denote significant differences (*P* < 0.05)

The concentration of sIgA in feces was lower in the HF diet sows than the LFM and LF diet sows on G110 d (*P* = 0.003; Fig. 3A). Interestingly, the concentration of sIgA in feces was increased from G110 d to L7 d (*P* < 0.001). The HF sows tended to have an increased concentration of sIgA (*P* = 0.057) on L7 d. We next detected ET and LCN-2 in plasma of sows on G110 d and L7 d (Fig. 3B and C). The results showed that sows from the HF and LFM diet groups had lower levels of ET than LF diet sows on G110 d and L7 d (*P* = 0.023 and 0.004, respectively). The HF diet group only had lower levels of LCN-2 than LF diet sows on L7 d (*P* = 0.009), and there was no significant difference in the HF and LFM diet groups. Furthermore, correlation analysis showed that the plasma concentration of ET on G110 d (*P* = 0.008) and ET and LCN-2 on L7 d (*P* = 0.037 and 0.021, respectively) were negatively correlated with the average daily feed intake during lactation (Table 7). In addition, a HF diet during gestation decreased the plasma TNF-α and IL-1β concentrations on G110 d (*P* = 0.019 and 0.022, respectively; Fig. 4A and B). The concentrations of plasma IL-6 and IL-10 were not different among the three groups (Fig. 4C and D).

**Table 7:** The correlations of ADFI of sows during lactation with the plasma hormone concentration

Items	Plasma (G110 d)		Plasma (L7 d)	
	ET	LCN-2	ET	LCN-2
ADFI of sows during lactation				
*r*	0.501	0.021	0.354	0.406
*R*^2^	0.251	0.001	0.126	0.165
*P*-value	0.008	0.915	0.037	0.021

*ADFI* Average daily feed intake, *G110 d* D 110 of gestation, *L7 d* D 7 of lactation, *ET* Endotoxin, *LCN-2* Lipocalin-2

### Concentration of SCFAs in sow feces

We observed changes in fecal SCFAs of sows on G110 d and L7 d (Table 8). There was a tendency for the concentrations of propionate (*P* = 0.092), butyrate (*P* = 0.084), and total SCFAs (*P* = 0.079) to be increased in feces of sows on a HF diet compared with sows in the LF diet group on G110 d. However, there was no significant difference in SCFA concentrations among the 3 groups on L7 d.

**Table 8:** Effects of high fiber diet or HF-FMT during gestation on fecal SCFAs concentrations of sows

Items	Treatment			SEM	*P*-value
	LF	LFM	HF		
110 d of gestation					
AA, μmol/g	42.46	45.77	50.01	2.919	0.177
PA, μmol/g	13.33	14.00	16.54	1.090	0.092
BA, μmol/g	6.44	7.59	8.01	0.584	0.084
SCFA, μmol/g	62.46	67.16	76.47	4.495	0.079
7 d of lactation					
AA, μmol/g	53.82	51.07	52.51	2.734	0.920
PA, μmol/g	18.58	15.99	15.69	1.018	0.516
BA, μmol/g	8.94	8.61	10.18	0.588	0.553
SCFA, μmol/g	77.14	82.58	75.68	4.122	0.790

Data were shown as least squares mean with their SEM. Values within a row with different superscripts differ (*P* < 0.05)

*LF* Low fiber diet during gestation, *n* = 15, *LFM* Low fiber diet + fecal microbiota transplantation from HF sow during gestation, *n* = 15, *HF* High fiber diet during gestation, *n* = 15, *SEM* Pooled standard error of means, *AA* Acetic acid, *PA* Propionate aid, *BA* Butyric acid, *SCFA* The sum of AA, PA and BA

### Characteristics of fecal microbiota community of sows

#### Difference in sow microbiota between G110 d and L7 d

**Fig. 5:**
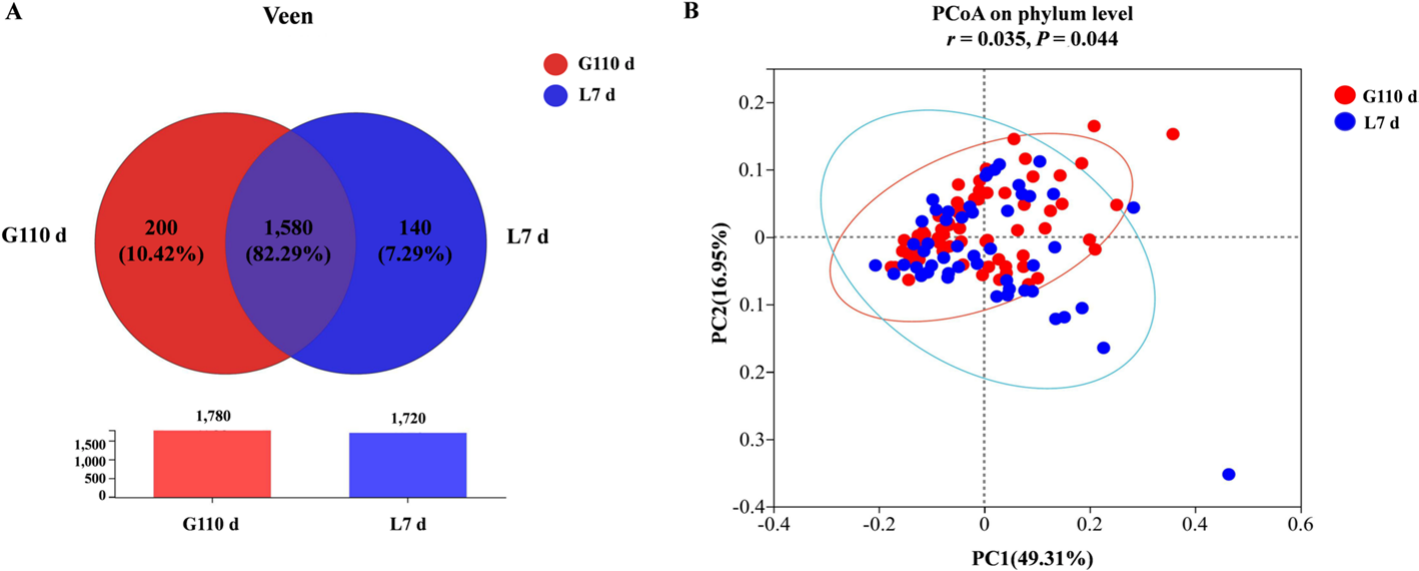
Comparative analysis of gut microbiota structure of sows on G110 d and L7 d. **A** Veen diagram of sows fecal microbiota on different day from late gestation to lactation. **B** Principal coordinate analysis of the gut microbiota communities of sows from late stage of gestation to lactation. LF, low fiber diet during gestation; LFM, low fiber diet + fecal microbiota transplantation from HF sow during gestation; HF, high fiber diet during gestation; G110 d, d 110 of gestation; L7 d, d 7 of lactation

As the Venn diagram shows (Fig. 5A), sows had common and special operational taxonomic units (OTUs) on G110 d and L7 d. Two hundred and 140 unique OTUs were identified in the G110 d and L7 d groups, respectively. G110 d and L7 d had 1,580 common OTUs. For beta diversity, the distribution of microbiota community at the two time points showed obvious characteristics of cluster along the principal coordinate, indicating that the composition of the microbial community shifted from G110 d to L7 d (Fig. 5B [ANOSIM]; *P* = 0.044).

#### Differences in sow microbiota among three treatments

**Fig. 6:**
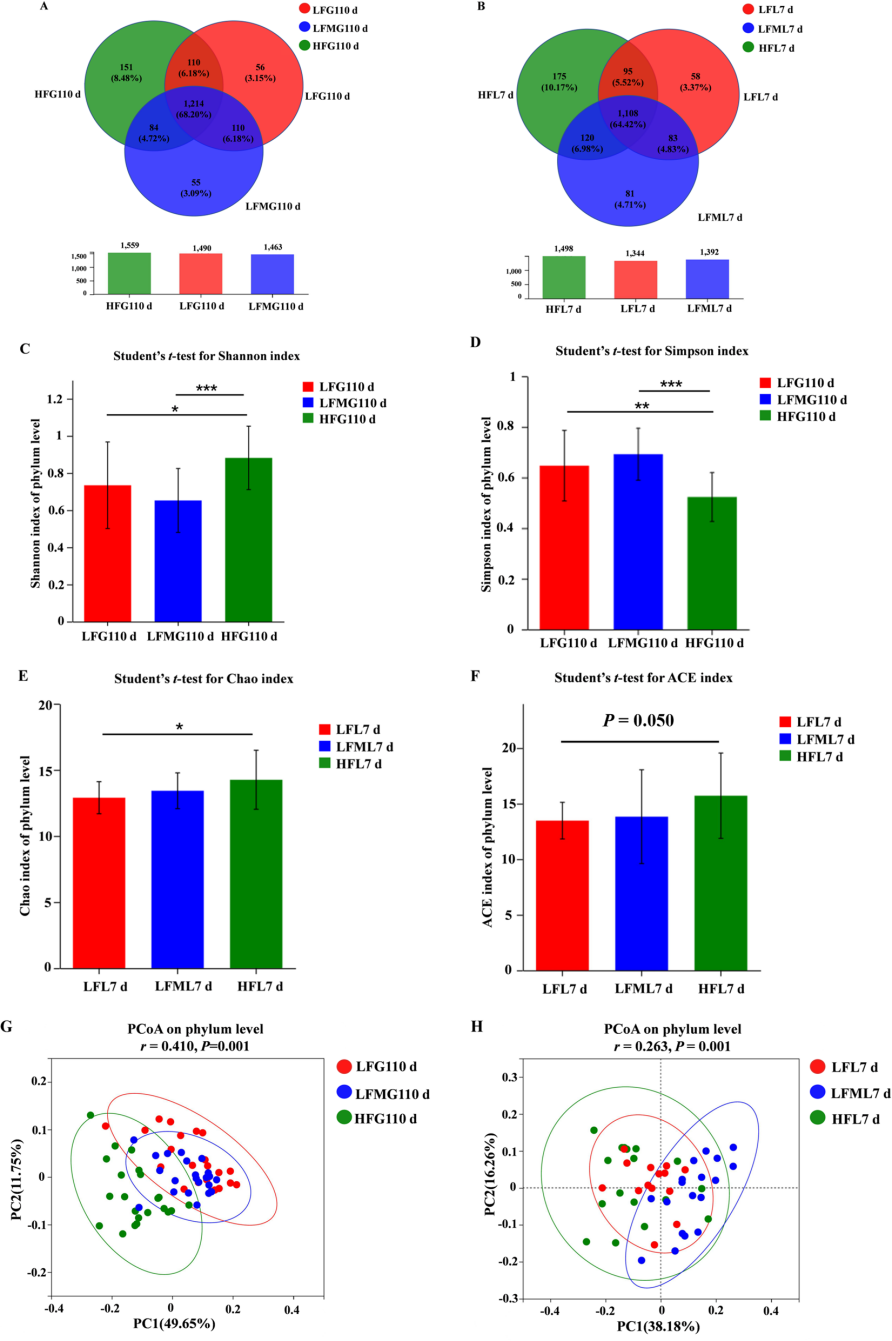
Effects of high fiber diet and HF-FMT during gestation on gut microbiota structure of sows. Veen diagram of sows fecal microbiota among three treatments on G110 d (**A**) and L7 d (**B**). Shannon (**C**) and Simpson (**D**) index of gut microbiota on G110 d, respectively. Chao (**E**) and ACE (**F**) index of gut microbiota on L7 d, respectively. Principal coordinate analysis of the gut microbiota communities of sows among three treatments on G110 d (**G**) and L7 d (**H**), respectively. LF, low fiber diet during gestation; LFM, low fiber diet + fecal microbiota transplantation from HF sow during gestation; HF, high fiber diet during gestation; G110 d, d 110 of gestation; L7 d, d 7 of lactation. G110 d: LF, *n* = 21; LFM, *n* = 23; HF, *n* = 22; L7 d: LF, *n* = 15; LFM, *n* = 18; HF, *n* = 19

As shown in Fig. 6A, each group exhibited unique OTUs and sows had their own special OTUs on G110 d. One hundred and fifty-one (~ 8.48% of the total OTUs) unique OTUs were identified in the HF diet group, 55 (~ 3.09% of the total OTUs) unique OTUs in the LFM diet group, and 56 (~ 3.15% of the total OTUs) unique OTUs in the LF diet group. These results were also observed on L7 d (Fig. 6B). One hundred and seventy-five (~ 10.17% of the total OTUs) unique OTUs were identified in the HF diet group, 81 (~ 4.71% of the total OTUs) unique OTUs in the LFM diet group, and 58 (~ 3.37% of the total OTUs) unique OTUs in the LF diet group. Furthermore, sows from the HF and LFM diet groups had a higher number of common OTUs than sows from the LF and LFM diet groups on L7 d (120 vs. 83, respectively). As shown in Fig. 6C and D, HF diet increased the shannon index and decreased the simpson index on G110 d. And sows in the HF diet group had higher chao and ace indices at the phylum level than the LF diet group sows on L7 d, but the LFM diet group sows only had a numerical increase. The beta diversity indicated that each group exhibited a separate composition of microbial community on G110 d and L7 d (ANOSIM; *P* = 0.001 and 0.001, respectively; Fig. 6E and F). Compared with the LF diet group, the composition of the microbial community in the LFM group was more similar to the HF diet group.

#### Changes in the relative abundance at the phylum level among the three treatments

**Fig. 7:**
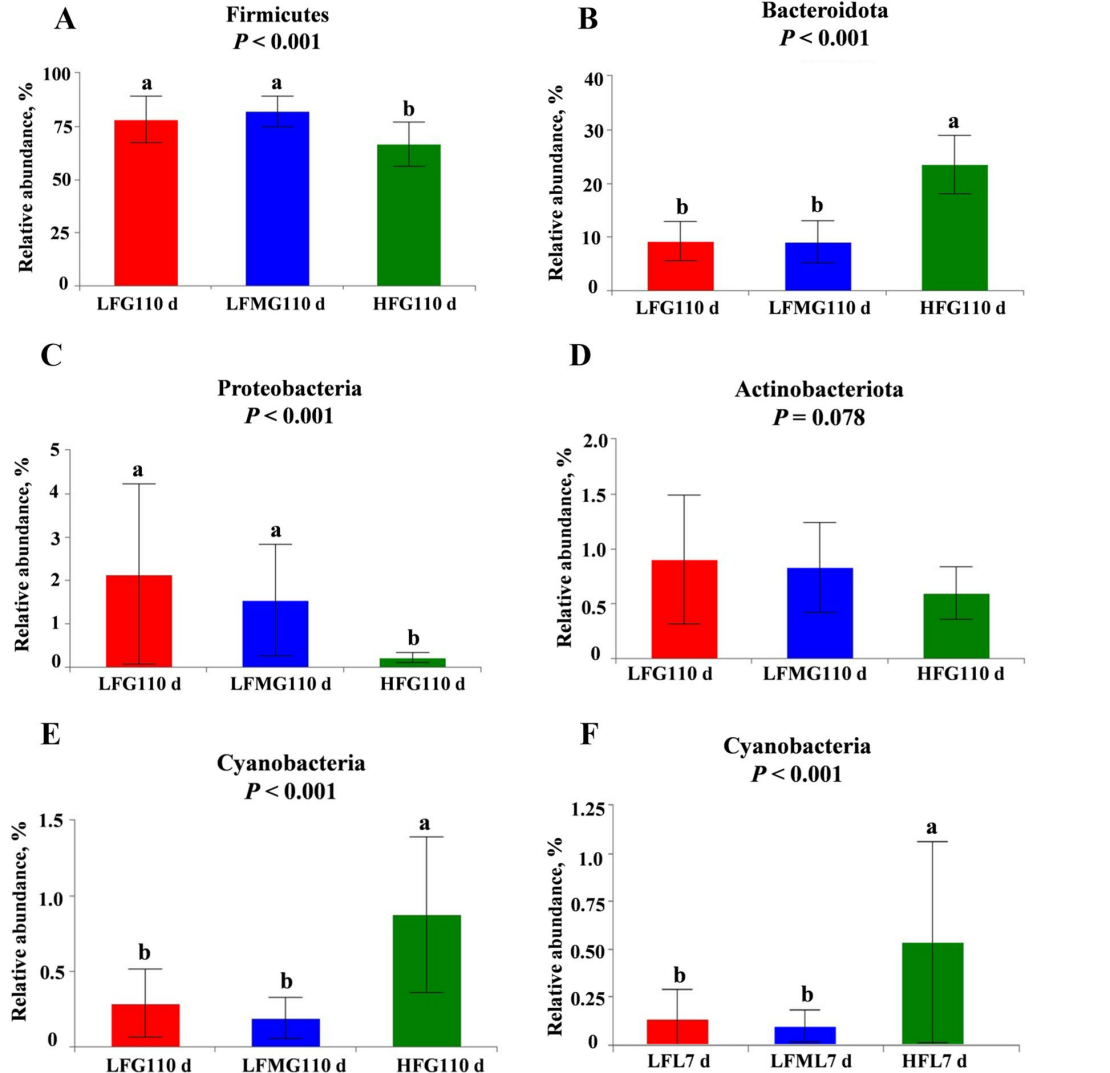
Effects of high fiber diet and HF-FMT during gestation on phylum level of gut microbiota among three groups on G110 d (**A–E**) and on L7 d (**F**). **A** Firmicutes, **B** Bacteroidota, **C** Proteobacteria, **D** Actinobacteriota, **E** Cyanobacteria on G110 d, **F** Cyanobacteria on L7 d. LF, low fiber diet during gestation; LFM, low fiber diet + fecal microbiota transplantation from HF sow during gestation; HF, high fiber diet during gestation; G110 d, d 110 of gestation; L7 d, d 7 of lactation. Data were expressed as means ± SEM. ^a,b^Different letters denote significant differences (*P* < 0.05). G110 d: LF, *n* = 21; LFM, *n* = 23; HF, *n* = 22; L7 d: LF, *n* = 15; LFM, *n* = 18; HF, *n* = 19

The changes in relative abundances at the phylum level of sows on G110 d and L7 d are presented in Fig. 7. On G110 d, HF treatment decreased the relative abundance of Firmicutes and Proteobacteria, increased the relative abundance of Bacteroidota and Cyanobacteria*,* and tended to decrease the abundance of Actinobacteriota compared with the LF diet group (Fig. 7A–E). Correlation analysis showed that abundance of Proteobacteria and Actinobacteriota on G110 d were negatively correlated with the average daily feed intake during lactation (Table 9). However, sows from the HF diet group only had a higher relative abundance of Cyanobacteria on L7 d (Fig. 7F).

**Table 9:** The correlations of ADFI of sows during lactation with the relative abundance of bacteria

Items	Feces, G110 d			Feces, L7 d		
	Actinobacteriota	Proteobacteria	*Escherichia-Shigella*	Actinobacteriota	Proteobacteria	*Escherichia-Shigella*
ADFI of sows during lactation						
*r*	−0.256	−0.245	−0.367	−0.106	−0.308	−0.309
*R*^2^	0.065	0.060	0.135	0.011	0.095	0.095
*P*-value	0.057	0.087	0.010	0.472	0.042	0.037

*ADFI* Average daily feed intake, *G110 d* D 110 of gestation, *L7 d* D 7 of lactation

#### Changes in the relative abundance at the genus and species levels among the three treatments

**Fig. 8:**
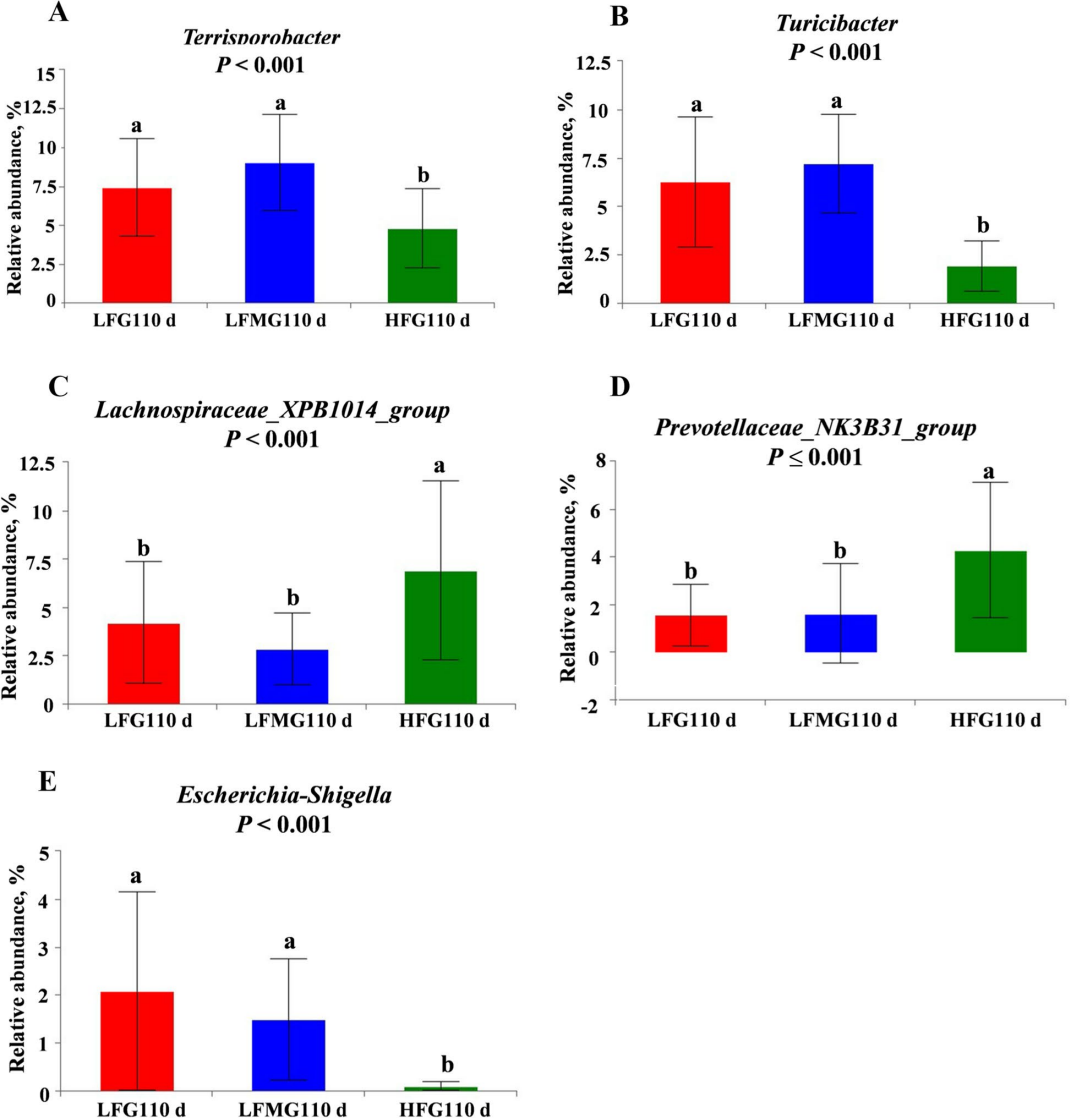
Effects of high fiber diet and HF-FMT during gestation on genus level of gut microbiota among three groups on G110 d. **A** *Terrisporobacter*, **B** *Turicibacter*, **C** *Lachnospiraceae_XPB1014_group*, **D** *Prevotellaceae_NK3B31_group*, **E** *Escherichia-Shigella*. LF, low fiber diet during gestation; LFM, low fiber diet + fecal microbiota transplantation from HF sow during gestation; HF, high fiber diet during gestation; G110 d, d 110 of gestation. Data were expressed as means ± SEM. ^a,b^Different letters denote significant differences (*P* < 0.05). LF, *n* = 21; LFM, *n* = 23; HF, *n* = 22

**Fig. 9:**
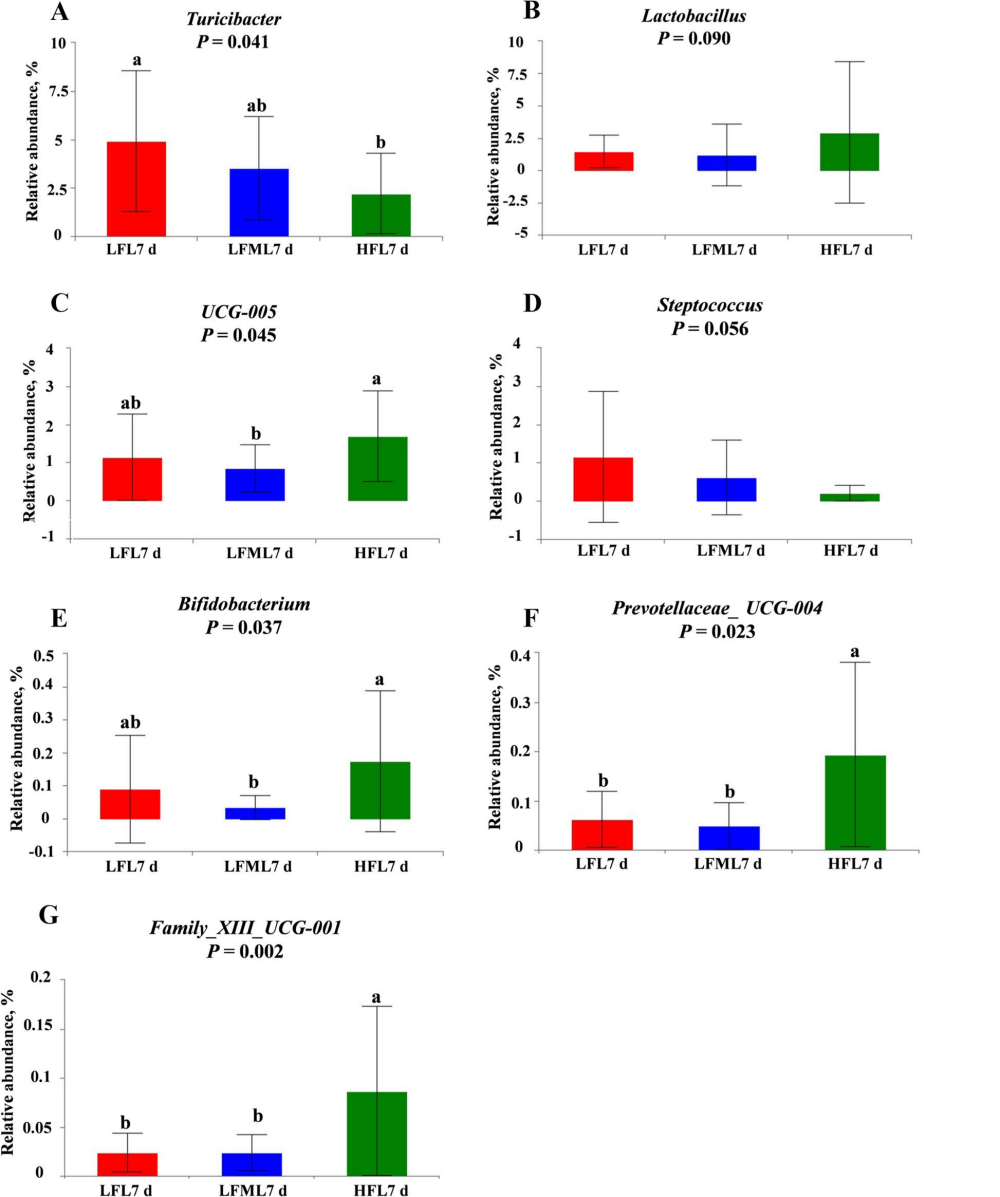
Effects of high fiber diet and HF-FMT during gestation on genus level of gut microbiota among three groups on L7 d. **A** *Turicibacter*, **B** *Lactobacillus*, **C** *UCG-005*, **D** *Streptococcus*, **E** *Bifidobacterium*, **F** *Prevotellaceae_UCG-004*, **G** *Familly_XIII_UCG-001*. LF, low fiber diet during gestation; LFM, low fiber diet + fecal microbiota transplantation from HF sow during gestation; HF, high fiber diet during gestation; L7 d, d 7 of lactation. Data were expressed as means ± SEM. ^a,b^Different letters denote significant differences (*P* < 0.05). LF, *n* = 15; LFM, *n* = 18; HF, *n* = 19

**Fig. 10:**
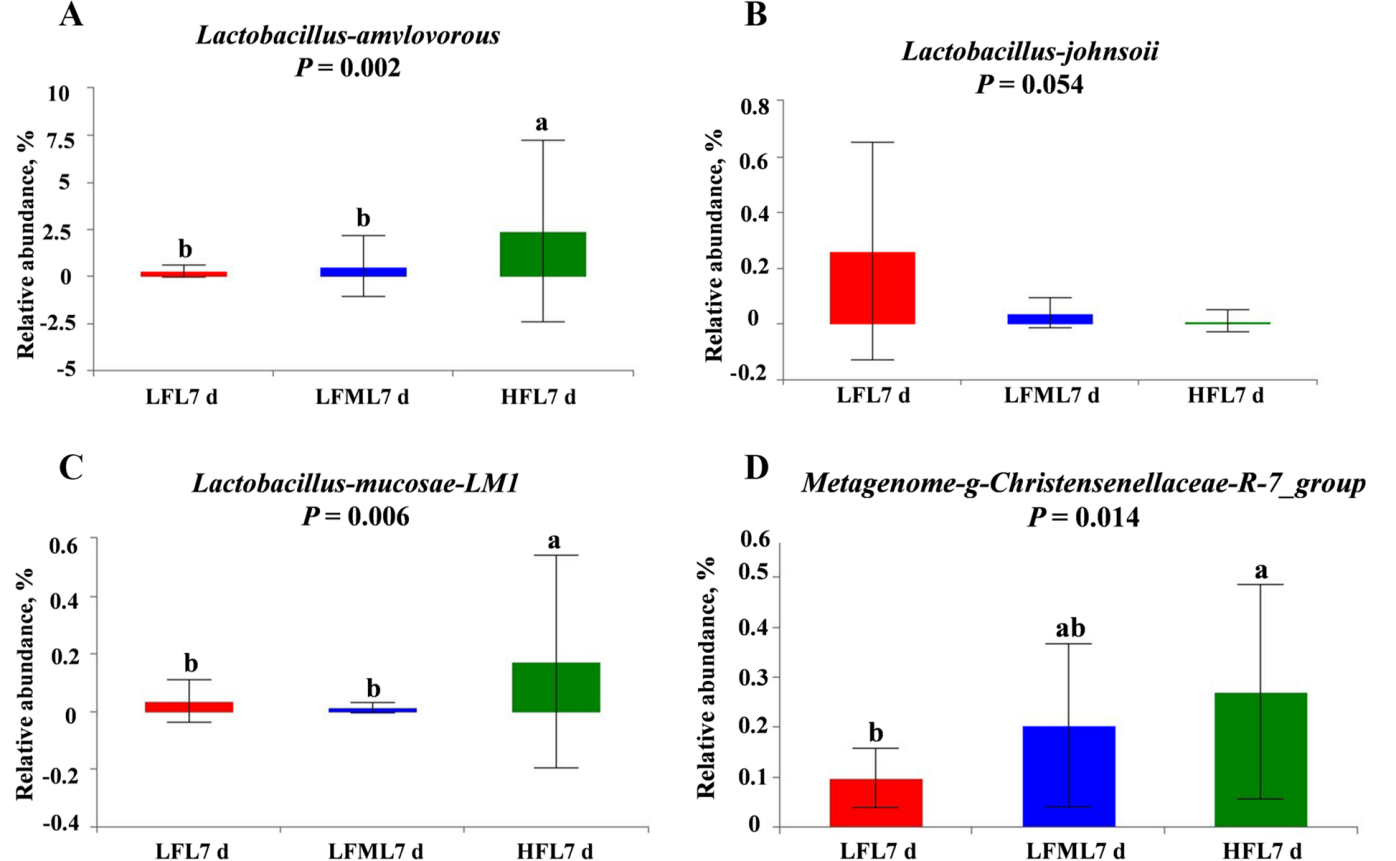
Effects of high fiber diet and HF-FMT during gestation on species level of gut microbiota among three groups on L7 d. **A** *Lactobacillus_amylovorous*, **B** *Lactobacillus_johnsoii*, **C** *Lactobacillus_mucosae-LM1*, **D** metagenome_*Christensenellaceae_R_7_group*. LF, low fiber diet during gestation; LFM, low fiber diet + fecal microbiota transplantation from HF sow during gestation; HF, high fiber diet during gestation; L7 d, d 7 of lactation. Data were expressed as means ± SEM. ^a,b^Different letters denote significant differences (*P* < 0.05). LF, *n* = 15; LFM,* n* = 18; HF, *n* = 19

The changes in relative abundances at the genus level are presented in Figs. 8 and 9. On G110 d (Fig. 8), the relative abundance of *Terrisporobacter*, *Turicibacter*, and *Escherichia-Shigella* were decreased, while the *Lachnospiraceae_XPB1014_group* and *Prevotellaceae_NK3B31_group* were increased by a HF diet during gestation. Correlation analysis showed that the abundance of *Escherichia-Shigella* on G110 d was negatively correlated with the average daily feed intake during lactation (Table 9). Compared with LF group, there was no significant difference in the abundance of *Escherichia-Shigella* between LFM and LF group, only a numerically decrease in LFM group (*P* > 0.05). On L7 d (Fig. 9A), the results showed that the abundance of genus *Turicibacter* was significantly decreased in the HF diet group (*P* = 0.041). The abundance of proinflammatory bacteria (*Turicibacter*) was alleviated in the LFM diet group (*P* > 0.05). *UCG_005* (*P* = 0.045, Fig. 9C), *Bifidobacterium* (*P* = 0.037, Fig. 9E), *Prevotellaceae_UCG-004* (*P* = 0.023, Fig. 9F), and *Family_ XIII_UCG-001* (*P* = 0.002, Fig. 9G) abundance at the genus level in the HF diet group sows were significantly higher than the LF diet group. Moreover, HF diet during gestation tend to increase *Lactobacillus* (*P* = 0.090, Fig. 9B) and decrease *Streptococcus* (*P* = 0.056, Fig. 9D) abundance. And *Lactobacillus_amylovorus* (*P* = 0.002, Fig. 10A), *Lactobacillus mucosae_LM1* (*P* = 0.006, Fig. 10C), and the *Christensenellaceae_R-7_group* (*P* = 0.014, Fig. 10D) at the species level in the HF diet group sows were significantly higher than the LF diet group. However, the abundance of *Lactobacillus johnsoii* tended to decrease in HF diet group than LF diet group (*P* = 0.054, Fig. 10B).

## Discussion

The lactation is an important period in the reproductive cycle of sows. And the feed intake during lactation is closely related to the reproductive performance of sows and the growth of piglets [26]. Our data indicate that high fiber diet or HF-FMT during gestation could improve the LFI of sows effectively. These results suggested that gut microbiota may be involved in the regulation of feed intake of sows during lactation.

In the present study, all sows consumed their daily feed completely throughout the entire gestation. As a result, sows ingested equal energy, crude protein, and fat content in each group. Therefore, there were no observed differences in BW and BF gain of sows during gestation. However, the HF diet during gestation tended to reduce the duration of sow parturition, which was consistent with the previous studies [27, 28]. Sows fed a HF diet during gestation consumed more feed than LF diet sows throughout lactation. Previous studies indicated that a HF diet (wheat bran, sugar beet pulp, and soybean hulls) during gestation increased the feed intake of sows similarly during lactation [4, 29]. At the same time, sows that received a HF diet FMT during gestation every day also increased feed intake during lactation. The offspring of HF and LFM diet sows had better growth performance during lactation, although the HF group did not achieve significant levels compared with the LF group. This finding consistent with a previous conclusion that LFI has the greatest effect on litter growth performance and sow subsequent productivity [26]. PYY and GLP-1 have been shown to be involved in satiety regulation [30–33]. These hormones affect satiety by reducing gut motility, delaying gastric emptying, and slowing transit time to enhance digestion and nutrient absorption, thereby reducing appetite [34]. In the current study, the HF and LFM diet groups increased the plasma PYY and GLP-1 concentrations on G110 d. These results indicated that a HF diet and HF diet FMT during gestation increased the plasma satiety hormone of pregnant sows, which contributed to relieving abnormal behavior and increasing postprandial satiety. These results were consistent with the previous studies that indicated DF supplementation during gestation increased the PYY and GLP-1 concentrations of pregnant sows and increased the LFI [31, 35]. Fetissov [36] indicated that non-digestible fiber was metabolized by bacteria produces several energy substrates. These bacteria-derived chemical signals activated the enteroendocrine cells to release PYY and GLP-1. Therefore, the fecal microbiota from sows in the HF diet group similarly increased PYY and GLP-1 concentrations in the LFM diet group. Moreover, our study also determined the plasma concentrations of PYY and GLP-1 during lactation. The data showed a HF diet during gestation increased these hormone levels on L7 d, and this finding was not observed in the LFM diet on L14 d. Few studies have observed the effect of a gestational HF diet on the concentrations of PYY and GLP-1 during lactation. There may be a carry-over effect of a fiber-rich diet offered during gestation on the behavior of sows during the first days postpartum cannot be ruled out. The increase in PYY and GLP-1 concentrations during late gestation and early lactation may be beneficial to voluntary feed intake of sows during lactation. These same results were observed in the HF and LFM diet groups, which suggested that a HF diet during gestation improves the feed intake during lactation by altering the gut microbiota of sows. Therefore, this study further analyzed the gut microbial composition of sows to reveal the role of gut microbiota in the regulation of feed intake during lactation.

In our study the results of 16S rRNA amplification sequencing revealed a clear shift in the gut microbiota structure from late gestation to lactation. This finding is consistent with previous studies that revealed the emergence of a dramatic change in gut microbiota of sows over the course of gestation and lactation [37, 38]. In addition, our data indicated that a significant difference in microbiota composition existed in the three groups. A lower abundance of Firmicutes and a higher abundance of Bacteroidota were observed in the HF diet group, which is consistent with previous studies [27, 39]. Bacteroidetes are well-known plant polysaccharide degraders [40]. A previous study indicated that the relative abundance of *Bacteroides_f_Bacteroidaceae* was significantly higher in sows with high litter performance. Bacteria in the family Christensenellaceae are known to be enriched in people with a low BMI [41] and are positively correlated with increased food intake and energy expenditure [42]. Liu et al. [38] indicated that the characteristics of Christensenellaceae were in agreement with the metabolic pattern (high food intake, energy expenditure, and body weight loss) of sows during lactation. Consistent with this study, the abundance of *Christensenellaceae_R-7_group* was increased on L7 d by a HF diet during gestation in our study. Moreover, the results also showed that a HF diet during gestation tended to increase the abundance of genus *Lactobacillus* and significantly raised the abundance of *Lactobacillus mucosae* and *Lactobacillus_amylovorus* on L7 d, while a HF diet increased the LFI at the same time. Secretory IgA is beneficial to the colonization of *Lactobacillus* in the gut [16] and is the major antibody in local mucosal immunity that can protect the intestinal epithelium from enteric toxins and pathogenic microorganisms [43]. In our study we showed that a HF diet tended to increase the concentration of sIgA in the feces. Therefore, enrichment of *Lactobacillus* may be related to an increase in sIgA in the HF group. The results were consistent with Shang et al. [44], who discovered that piglets from wheat bran-fed sows had the highest sIgA concentration and a high abundance of Lactobacillaceae. *Lactobacillus* are important probiotic bacteria in the gut, and some studies have shown that *Lactobacillus* can help to maintain the homeostasis of gut microbiota. In addition, an oregano essential oil diet enhanced the fecal *Lactobacillus* of sows and had a tendency to enhance feed intake of sows in the third week of lactation [45]. Tibetan pig-derived probiotic *Lactobacillus amylovorus* SLZX20-1 improved the weight gain and average daily feed intake of mice [46]. Tan et al. [30] reported that supplementation of soluble fiber in gestation diets increased the abundance of *Lactobacillus* and improved the feed intake of sows during lactation. Bagarolli et al. [47] showed that *Lactobacillus* supplementation improved insulin resistance and low-grade inflammation caused by a HF diet in mice. A previous study showed that insulin insensitivity is detrimental to sow lactational feed intake [48]. Some studies have shown that lean sows eat more feed during lactation than fat sows [49] and the reduced ADFI is caused by greater insulin resistance [50, 51]. The decrease in LFI of sows may be caused by excessive reduction of insulin sensitivity in late gestation and early lactation [52]. Enrichment of *Lactobacillus* may be beneficial to the feed intake of sows during lactation by improving insulin resistance. In recent years, some studies have examined the health-promoting properties of *Lactobacillus johnsonii* on humans, sows, and mice [53, 54]. Chagwedera et al. [16] reported that activation of mTORC1 in CD11c cells decreased food intake and body weight in lean mice. Surprisingly, the transplantation of *L. johnsonii* Q1-7 alleviated this phenomenon, suggesting the existence of transkingdom immune-microbiota circuits for homeostatic regulation of food intake and body mass in healthy mice. However, the abundance of *Lactobacillus johnsonii* was higher in the LF diet group, which had low LFI in our study. This result was consistent with another study that found use of the strain, *Lactobacillus johnsonii* XS4, from d 90 of gestation to d 25 of lactation was beneficial to sow production performance, but had no effect on feed intake of sows during lactation [55]. This finding could be due to the dosage of the *Lactobacillus* and the stage of supplementation. Furthermore, the specific species and even the strains of *Lactobacilli* beneficial to the feed intake of sows during lactation need further study.

In addition, the results showed that the abundance of Proteobacteria and *Escherichia-Shigella* was higher in the LF diet group on G110 d. Proteobacteria is a tiny component within a balanced gut microbiota [56]. However, during the past few years, many studies have suggested that an expansion of the potential diagnostic microbiologic signature of imbalanced gut microbiota, gut inflammation, and epithelial dysfunction is an extension of Proteobacteria [57]. Similarly, Proteobacteria are increased in women during late pregnancy and can cause insulin insensitivity and inflammatory responses in germ-free mice by FMT [58]. A previous study found the proportion of Proteobacteria in gut microbiota sows was higher in late gestation [59]. Our data showed that the feed intake of sows during lactation was negatively correlation with the abundance of *Escherichia-Shigella* on G110 d and L7 d. And the feed intake of sows during lactation tended to be negatively correlation with the abundance of Proteobacteria on G110 d. Clearly, these changes may have deleterious effects on host metabolism because Proteobacteria and *Escherichia-Shigella* are often related to inflammatory conditions and insulin insensitivity. The addition of HF during gestation decreased the enrichment of Proteobacteria and *Escherichia-Shigella*. Therefore, the results indicated that the lower Proteobacteria and *Escherichia-Shigella* in the HF diet group on G110 d was beneficial and increased the feed intake of sows during lactation. There was no significant reduction of Proteobacteria and *Escherichia-Shigella* abundance by FMT, but the enrichment in G110 d was mitigated by FMT. The beta diversity indicated that the composition of gut microbiota in the LFM diet group was more similar to the HF diet group. Furthermore, phylum Proteobacteria and genus *Escherichia-Shigella,* which belong to gram-negative bacteria, produce lipopolysaccharide (LPS). An increase in LPS activates the TLR4 signaling pathway to cause insulin resistance [60]. Once bacterial LPS entering the circulation through the gut barrier is decreased, the concentration of bacterial endotoxins in the circulation increases, which is a potential mediator of inflammation causing metabolic endotoxemia [61]. Similar results were observed in our study. Specifically, a HF diet and FMT during gestation decreased the concentration of plasma ET, which is a biomarker of gut permeability on G110 d and reduced the level of plasma LCN-2 on L7 d. LCN-2 is a neutrophil protein that binds bacterial siderophores and is associated with low-grade inflammation [62]. This finding was consistent with a recent study that indicated dysbiosis in gut microbiota may increase gut permeability [63–65]. Moreover, our data showed that the feed intake of sows during lactation was negatively correlated with the plasma ET concentration on G110 d and L7 d and plasma LCN-2 only on L7 d. In addition, our data showed that HF increased the α-diversity of gut microbiota on G110 d and L7d. A previous study showed that low gut microbiota richness is associated with increased gut permeability in overweight pregnant women [66]. There is a correlation between low gut microbiota richness and adverse metabolic conditions, such as a more pronounced inflammatory phenotype [67].

Therefore, enrichment of Proteobacteria and *Escherichia-Shigella* and the decrease in gut microbiota richness and *Lactobacillus* in the LF diet group may be the reason of the inflammatory phenotype of sows in our study. A HF diet during gestation decreased the pro-inflammatory cytokine concentration of TNF-α and IL-1β on G110 d. In agreement with our study, these previous studies showed that the addition of insoluble fiber (wheat bran or lignocellulose) decrease the IL-1β and TNF-α gene expression levels in the ileum [44]. Johnson [68] suggested the TNF-α induced anorexia behavior of hosts. Liu et al. [69] pointed out that adding dietary fiber (alfalfa meal) during gestation reduces the concentration of serum endotoxin and TNF-α, and increases food intake during lactation of sows. The current study indicated that *Terrisporobacter* was positively correlated with serum lipocalin-2, TNF-α, and endotoxin. Munyaka indicated that enrichment of *Terrisporobacter* may cause colitis to promote gut microbiota malnutrition in animals. A decrease in *Terrisporobacter* abundance was observed in the HF diet group on G110 d and L7 d. A sugar beet pulp diet during gestation increased *Lactobacillus* abundance and decreased *Terrisporobacter* abundance of sows on G110 d and L7 d, and decreased the TNF-α of sows at the same time [70]. In addition, a HF diet tended to decrease the abundance of Actinobacteria in our study, which has been shown to be substantially more abundant in inflammatory bowel disease patients [71]. Zhou et al. [72] indicated that the use of soluble DF inulin tended to decrease the abundance of Actinobacteria*,* while improving the inflammatory response of sows during the perinatal period. These findings indicated that a HF diet or HF diet FMT during gestation improved LFI by alleviating the inflammatory status of sows in late gestation. The mechanism was likely via an increase in the abundance of *Lactobacilli,* especially in species *Lactobacillus mucosae* and *Lactobacillus_amylovorus* on L7 d and inhibiting the abundance of Proteobacteria and genus *Escherichia-Shigella.*

SCFAs are the main products of gut microbial fermentation [73]. In our study a tendency was observed only in the HF diet group to increase the concentrations of PA, BA, and total SCFAs. Ruminococcaceae, Clostridiaceae, *Prevotella,* and Lachnospiraceae bacteria encode a large number of carbohydrate-degrading enzymes and are known producers of SCFA [74, 75]. Thus, it is not surprising that increased abundance of *Lachnospiraceae_XPB1014_group* and *Prevotellaceae_NK3B31_group* belongs to Clostridiaceae and *Prevotella* was observed on G110 d in the current study. SCFAs can also participate in long-term regulation of energy metabolism. A previous study indicated that systemic administration of butyrate and propionate stimulated intestinal gluconeogenesis, which could increase satiety in the host. Therefore, an increase in SCFAs would be beneficial to satiety in pregnant sows.

## Conclusion

The current study showed that the gut microbiota of sows changed dramatically from late gestation to lactation, and sows with high and low feed intake during lactation had a unique microbial community. The use of a HF diet during gestation could increase the feed intake of sows during lactation by increasing the abundance of *Lactobacilli,* especially *Lactobacillus mucosae* and *Lactobacillus_amylovorus* on L7 d, and inhibiting the abundance of Proteobacteria and genus *Escherichia-Shigella* on G110 d*.* The changes in these bacteria could relieve systemic inflammation of sows. Furthermore, sows that received a HF-FMT during gestation had similar results (such as performance during lactation, inflammatory state etc.) with sows in the HF diet group. These results indicate that gut microbiota play an important role in feed intake regulation of sows during lactation. Our study provides novel insights to promote the LFI of sows by targeting a change in the gut microbiota.

## Data Availability

The datasets analyzed in the current study are available from the corresponding author on reasonable request.

## Abbreviations

AA: Acetic acid
ADFI: Average daily feed intake
ADG: Average daily gain
ATTD: Apparent total tract digestibility
BA: Butyric acid
BF: Backfat
BW: Body weight
CP: Crude protein
DF: Dietary fiber
EECs: Enteroendocrine cells
ET: Endotoxin
FMT: Fecal microbial transplantation
GLP-1: Glucagon-like peptide 1
HF: High fiber
IL-1β: Interleukin-1β
LCN-2: Lipocalin-2
LF: Low fiber
LFI: Lactation feed intake
LPS: Lipopolysaccharide
NE: Net energy
OTUs: Operational taxonomic units
PA: Propionic acid
PCoA: Principal coordinates analysis
PYY: Peptide tyrosine tyrosine
RDP: Ribosomal Database Project
SCFAs: Short-chain fatty acids
TNF-α: Tumor necrosis factor-α
